# Ideological representation of fear and hope in online newspaper reports on COVID-19 in Saudi Arabia

**DOI:** 10.1016/j.heliyon.2021.e06864

**Published:** 2021-04-28

**Authors:** Naimah Ahmad Al-Ghamdi

**Affiliations:** Department of English, College of Arts, Imam Abdulrahman Bin Faisal University, Dammam, Saudi Arabia

**Keywords:** Ideology, Fear, Hope, Covid-19, News reports, Saudi Arabia

## Abstract

This study examines the ideological representation of fear and hope in online newspaper reports on COVID-19 in Saudi Arabia. The study adopts critical discourse analysis (CDA) of Van Dijk's tripartite framework of news text structure and production at microstructure level and Fairclough's insights on the interactivity between language and social cognition in the process of producing and interpreting texts. The data for this study is samples of online news on COVID-19 reported in two English Saudi website;Saudi Gazette and Arab News, six from each journal. The samples cover the period between March 4 and August 14, 2020, and are purposefully chosen to understand the language of the news reports on a specific issue over a period of time. A qualitative analysis of the data is carried out so that the perceived effect of the linguistic encoding of the news events is examined. The findings show that most of the reports on COVID-19 heavily rely on foregrounding evidentiality of statistics of new and cumulative infections as well as recoveries and deaths. Evidentiality strategy often overlaps with categorization strategy as proof of the threat of the virus which is broken down into units, each with its features and evidence. Authority strategy is used to support statistics with verifiable sources. Similarly, in representing hope to the populace, two strategies of evidentiality and authority are often used. Evidentiality strategy is employed to clear off doubts through for grounding statistics of growing recoveries, comparisons and underrepresentation of infection cases and mortalities and sometimes choice of verbs plays a role in imparting hope in the residents. Authority strategy is deployed in order to give some weight to the ideology inherent in the report.

## Teaser

Ideology; Fear; Hope; Covid-19; Newspaper; Saudi Arabia.

## 1 Introduction

With the breakout of Coronavirus (Covid-19) in China in December 2019 and the subsequent discovery of the first case in Saudi Arabia on March 2/2020, a wave of panic pervaded the scene. The fear was grave real as people witnessed unprecedented measures in all aspects of their daily lives. Saudi Arabia suspended all flights, religious seasons (Umrah and Hajj) was postponed and bar citizens and non-citizens from entry to the country and later ease restrictions for non-citizens. The social media and the daily updates of the global and local news confirmed the deadly nature of the virus and infected cases and fatalities mounted day after another. Since its occurrence, there have been different reactions to its discourse. These reactions range from governmental, institutional, political to medical, personal and regional discourse domains. Being a threat to the existence of humanity, a lot of reactions to it have been those that admonish precaution to it and the ones that proffer suggestive solutions. These reactions are mediated by the media agencies, a development that may introduce the ideological underpinnings into the report.

Emotions of fear and hope in this study are a cognitive experiences of the mind which can be created by the public community in Saudi Arabia when reading these on-line news reports. Such cognitive experience of the mind can be triggered through the way texts ideologically reconstruct certain strategies, for instance, evidentiality, categorization of the infected as “Saudi/non-Saudi, expats”, attributing statements to authorities reliable institutional or senior figures or general sources. The study claims that, at the macrostructure level of texts, foregrounding and backgrounding reports of Covid-19 news, ordering the information of death trolls, critical cases, new cases, recoveries, and sometimes choices of verbs are conductive of fear and hope of the community. Microstructures of discourse are derived or inferred from the local meanings of (words, phrases, sentences and connection between them, “text grammar” as used by Van Dijk (1980:38) of discourse by the aforementioned strategies that redesign facts into reports about facts.

Media discourse should be conceptualized in terms of its context structures (Duranti & Goodwin inVan Dijk, 2006), Van Dijk (1998b). News reports, in particular, have their own particular structure which corresponds to information gradation consisting of headlines, the byline “who wrote the report”, the main lead, the body and an end. This is what Van Dijk (1998b) called the “thematic structure” of a news topic. News reports have their political, social and ideological norms and values which represent the ideological stance of the news agency or the news editor. They report new news, inform and update by retelling and redesigning information about a significant worldwide or contemporary event. Besides News has its structures in connection to the functions they have when they are enacting news information and processes, such as governing, legislating and with very specific aims in mind, such as alerting, evaluating or instructing. The news agency obviously does not participate mindlessly in such situations but have socio-political knowledge, share political norms and values, as well as certain ideologies, Van Dijk (2006). Indeed, it is through this form of contextualization that we can link the ideologies of the participants to their discourses as supported by Richardson (2007), Catenaccio et al. (2011) Like text or talk, news reports show ideologies discursively in connection to the copywriters, editors and news agencies. Media reports are significant as it has the power to orient public opinion and foreground certain figures and background others to “create a negative or positive influence and reaction among readers especially that their well-being, and safety are under threat” (AlAfnan, 2020). This is what the present study undertakes to unfold the enactment of ideology in such texts through meaning-making of the microstructure employed in news reports of Covid-19.

## 2 News and representation

White in Post (2008) supports the view that a discursive analysis is one in which “social participants have been chosen for inclusion in a text and which participant roles they have been chosen for consistently within the text are a valuable tool for discovering ideological positioning that is not only evaluative but covert” (142). Stubbs (1983) believes that language potential and its socio-political and cultural manifestation in a text can be understood through the examination of how language is deployed within the text. Stubbs, like Wodak in Van Dijk (2006) believed that linguistic strategies in a news discourse act like" plans of action that may vary in their stylistic elaborations, effect and might be un/consciously sought contrary to its factual and linguistic realizations in reality.

The position that texts are interactive and representative has been repeatedly expressed by (Catenaccio et al., 2011; Van Leeuwen, 2008; Van Dijk, 1993). Van Dijk is particularly of the opinion that text is a form of knowledge control, and knowledge control in itself has the tendency to influence people's interpretation, attitudes and actions (Van Dijk, 1993). For instance, people's behavioural patterns during COVID-19 crisis are affected by news contents. In other words, what the majority of people know about the pandemic is largely dictated and linguistically redesigned or foregrounded or by news texts.

Fairclough (1992), perceived the social interactivity between language and social cognition in the process of producing and interpreting texts as forms of social practice conditioned by linguistic and non-linguistic (cultural, economic, political and affective etc) elements. Media texts, whether spoken or written, are forms of social discourse in action and the CDA tools play a significant rule in explaining the structures of social and political issues within discourse, it is able to expound upon the various orders of language within discourse by deconstructing the methods used to maintain hegemony by elite groups via their use and influence of media to further their own ideological dominance over individual lives within societies (Van Dijk, 2001 in Post 2008). Thus, such a discourse not only presents a reflection of the social order as perceived by individuals within society, but also shapes the social order of individuals’ interactions with society.

## 3 Language and ideology

Ideologies are essentially fundamental cognitions of a social group, such as socialists and politicians and people in the press who underlie their attitudes and indirectly their discourses and other social practices. According to Van Dijk, 1993, Van Dijk, 1995, Van Dijk, 1998a, Van Dijk, 1998b, ideology binds the tripartite of social cognition, discourse and society together. An investigation of ideological strategies in discourse can reveal an interpretive channel to the relationship between society and language. Thus, social structures such as manipulations, legitimation of power and dominance, concealment of social actors or recipients of action, are reflected in discourse. While Van Dijk adopts a discoursal approach to ideology representation, Hart (2016) adopts a functional cognitive perspective by addressing the socio-cognitive dimensions of CDA and ideology. He notes that the Cognitive Linguistic Approach (CLA) emphasizes the relationship between text and conceptualization in contexts of social and political discourse. It is this conceptualization that is involved in meaning construction as language connects with the background knowledge to yield local mental representations. Such conceptualization takes up different forms (metaphors or mappings) and CLA theorizes analytically the effects of conceptual ideological language on linguistic choices, cognitive grammar and even metaphors. Thus, language choices become functional of social discourse ideology and social action.

In explaining the controlling power of discourse, Van Dijk (1995: 244) uses the term “discourse access”. This control is characterized by the unique situation and the structures of a text. The structure of discourse can be manipulated so that it defines discourse access. Form, meaning and style can be manipulated or controlled through such linguistic techniques as grammatical changes and rewording. Fairclough (1995: 27) identifies these as “ideological-linguistic processes”. Apart from these techniques, passivization, nominalization/topicalization, lexicalization, layering or foregrounding can also be explored in a discourse. The control of the structure of discourse is indexical to the “hidden power” of discourse (Fairclough, 1989: 35).

Discourse structure, discourse agents and context play important roles in influencing mental perceptions or representations. If discourse is controlled, according to Van Dijk (2006), then mind control will be easier to achieve, and individuals have a tendency to acknowledge as genuine or true opinions, beliefs and knowledge from sources felt to be reliable such as experts, professionals, scholars, or the mass media (Van Dijk 2001: 358) particularly when they have no means of verifying the information. Thus, it is this power behind discourse that represent the ideologies of their reporters and agencies concerning what kind of information to give to the readers and how to order and construct them, what sources to trust, use and cite that trigger attitudes, actions and sentiments.

Ideology representations is an important subject in CDA. It is characterized as the interface between social cognition and social structure and as the mental frameworks–the languages, the concepts, categories, imagery of thought, and the systems of representation” (Morley and Chen 1996: 25) that different individuals and groups use to comprehend and clarify different social practices. More importantly, ideologies serve as symbolic systems of thought to organize, stabilize and maintain particular forms of power relations. In CDA, there is an attempt to identify the ideologies (explicit and implicit types) in any text and to bring them to the attention of the reader. Caldas- Coulthard in Post (2008:9) states, “all texts code the ideological position [s] of their producers” and it is through CDA that meaning-making of such ideologies is attained.

Several scholars such as Lemke and Halliday have observed that language users often manipulate language in their manifestation or exhibition of their ideology. For Lemke (1995) specifically, this offers an individual an opportunity to shape meaning towards a certain line of thought or belief. To Halliday, reality is constructed according to the predisposition and experiences of the language user and this is reflected in the way s/he builds her/his functional grammar. In other words, the language user is solely responsible for any ideological traits found in grammar construction (Stubbs in Post (2008). This view is also in consonance with that of Van Dijk (1995: 248) who asserts that ideologies are products of “relevant sociocultural values… (which are) organized by an ideological schema that represents the definition of a group”. To Van Dijk (1995), every analysis of discourse is invariably an analysis of ideology. This is because ideologies are often “expressed and reproduced” in written, spoken and pictorial texts. And that is why, for him, an analysis of ideology would focus on any of (or all the) these three: social analysis, cognitive analysis and discourse analysis (Van Dijk, 1995). In other words, an analysis of ideology must be grounded within the social consciousness of a language user. That is why Chouliaraki and Fairclough (1999: 6) view CDA as a field that blends linguistics with social science “within a single theoretical and analytical framework, setting up a dialogue between them”.

In his enactment of ideology in discourses and texts, Van Dijk (1993; Van Dijk, 1998a, Van Dijk, 1998b) discussed the media texts in terms of structure, production and comprehension processes. These three processes are not independent because they are related with the social context where news item occurs. This relatedness can manifest at micro and macrostructure. The former has to do with the relationship between various construction elements, such as syntax, lexis and rhetoric, in the news text and those elements that adduce some evidentiality or factuality to the text. The latter relates to the thematic structure of the text and its overall schemata.

Van Dijk (2001) highlights a number of categories that suggest the manner in which ideologies may manifest. For instance, **Actor Description represents** the way in which a speaker or writer's ideology could be depicted through the way they describe actors in an event. For instance, certain group of people may be described in a positive or neutral way if they are members of the in-group while others may be described in a negative way if they belong to the out-group, while **Generalization** is often employed in a racist discourse such that prejudices and negative characteristics are over-generalized. Ideology may also be depicted through the strategy of **Authority**, **Categorization** and **evidentiality**: authorities are often cited by people from different ideologies in order to give weight to their argument; people are often categorized along their social status, ethnicity, religion or race; and at times, people compare poor situations or activities with others in order to offer justification for some. For instance, poorly managed economies are compared with one another in order to declare one better or worse than the other. **Evidentiality** involves backing up certain lines of argument often with cited figures or statistics and numbers. There can also be the use of **Disclaimers, Euphemism** in constructing one or more ideology.

## 4 Theoretical framework

The study adopts Van Dijk, 1993, Van Dijk, 1998b CDA theory in relation to three strategies *authority, evidentiality and categorization* that feature ideologies related to fear and hope in Saudi news reports within the country in 2020. Textual microstructure analysis of news reports is employed in the representation of Saudi covid-19 texts.

## 5 Research questions

The present study attempt to answer the following question:

1: How Covid-19 news reports ideologically represent hope and fear in selected daily Saudi Gazette and Arab News; what are the strategies employed to do so?

## 6 Review of literature

The discourse on Covid-19 has been a subject of extensive investigation particularly in medicine, health and educational field researches compared to the relatively limited research on linguistics analysis about Covid-19 in the media. Discourse on Covid-19 have been carried out in the social media providing insights of communities sociolinguistic practices during the pandemic (Al-Ghamdi and Albawardi (2020); Nugrahat et al. (2020), in relation to thematic variation of the residential care covid-19 discouse (Allen and Ayalon (2021), in cognitive belief system surveying how far individuals believe in Covid-19 Conspiracy Theory Miller (2020), in presidential speeches investigating the interplay between language and power (Olimat (2020) and in the manipulative language of the press (Abbas, 2020; Rafi, 2020). Moreover, news discourse has been widely studied from a discursive structural perspective and from a transdisciplinary perspective stimulating the debate on the linguistics, socio-political, cultural and economic functions of journalism Catenaccio et al. (2011), the integration of CDA in a discursive and social analysis of news reports as advocated by Richardson's (2007) form of the relations between discourse and news ideologies such as the use of biased lexical items, syntactic structures such as actives and passives, pronouns such as us and them, metaphors, arguments, implications, and many other properties of discourse.

Focusing on the ideological construction of the Gulf crisis in Arab media discourse Kharbach (2020), presents a textual and socio-cognitive of analysis of the media communication discourse. The researcher adopted a CDA of Van Dijk to discursively construct the subjective attitude of Aljazzera &Alarabia news articles. The study is informative for the current study in highlighting the role of semantic-syntactic aspects of headlines in revealing the ideological stances of these two news channels is specifically centered on the ideological construction of the Gulf crisis in Arab media discourse from a textual and socio-cognitive levels of analysis. The present study would advance research on the ideological experimentation of language in Saudi Arabia by examining how fear or hope are constructed in online news reports on COVID-19. Alsaedi (2021) traces the metaphorical representation of fear in two English Saudi newspapers (two from Saudi Gazette and four from Arab News)adopting cognitive metaphor theory and Pragglejaz Group methodology and concluded that these newspapers rely heavily on war metaphors, Sinophobia metaphors, and metaphors allaying fears in writing about Coronavirus Crisis.

In two articles from two leading American and Chinese Newspapers, AlAfnan (2020) employing a CDA approach; contrasted the linguistic strategies of bias and dominance implicitly assonating media ideologies of the writers or the news agency via foregrounding and backgrounding of information and ideas. Nor et al. (2020) downloaded online newspaper reports about Covid-19 from March 1st to March 31st, 2020 and processed and analyzed a corpus of 1018 news reports were classified into 140 themes being 100 top collocates with the MI score using AntConc software revealed that verb and noun collocates co-occurred frequently with the node word Covid-19. These noun/verb collocates reflect the majority of Malaysians expressions of fear, anxiety and uncertainty.

## 7 Methodology of the study

Covid-19 pandemic caused unprecedented global panic and the situation in the Saudi community was no less tough and it cling the social media, the press and the people busy in daily news and updates about covid-19. The present study is interested on how Saudi online news reconstruct Covid-19 textually to orient fear or hope to the public at home; Gulf Arabic English speaking citizens as well as the multi-nationalities residents in Saudi Arabia. Moreover, the study fills in the gab in relation to the examination of the microstructure text analysis of Covid-10 in digital news reports which is not investigated yet. Saudi Gazette and Arab News journals report the views of their Saudi agencies and sometimes that of the writer. The two English newspapers are issued from Saudi Arabia. Saudi Gazette started publication in 1976 while the Arab News in 1975. This study is not ment to be a comparative study of the ideological stances of these two news journals, but rather on how, with the aid of CDA, the microstructure of Covid-19 news reports function to give negative or positive orientations to its articulation. Moreover, headlines of the news reports are beyond the scope of the present study as some of the headlines integrate semiotic dimensions.

The data for this study is purposefully obtained from news items on COVID-19 reported in two Saudi online newspapers: Saudi Gazette and Arab News, six texts from each journal that come under COVID-19 Daily Reports in Saudi, irrespective of their emotive appeals (See Table I the data of the study in the Appendix 1, for the complete news reports which uptake fear or hope invoked experiences in the two assigned journals). The selection of these items, covers the period between March 4 and August 14, 2020, is based on the effort to probe into the role of the news reports in reconstructing the discourse about COVID-19 to intentionally create a cognitive experience of hope and fear in the reader. The study is, therefore, a linguistic input to this effort since the study demonstrates certain explicit or implicit ideology underlying the news reports of the pandemic in Saudi Arabia. A qualitative microstructure method of analysis in line with Van Dijk's CDA and Fairclough insights is carried in this study. The samples were taken from the Saudi news websites and are representative of the focus of the study Covid-19 reports from March till August seemed adequate and are homogeneous in thematic content for understanding the informative language of the news reports on a specific issue over a period of time.

The data analysis and discussion section is qualitative in nature and is divided into two sections, the first lay out 7 news reports from Saudi Gazette and Arab News motivating fear and the second section lay out 5 news reports from the two newspapers generating hope appeal. The news reports are arranged in a chronological order of occurrence, citing the source and the writer name when available. Ideological representation of fear is first handled then hope ideology is examined. This order of news reports is informed by the nature that COVID-19 reports sequence in Saudi; early reports replete with fear and panic while the later ones come with hope as the pandemic gets curbed day after another and the severity of the situation is reduced. Data analysis of the news reports are presented in line with the theoretical framework highlighting the linguistic strategies adopted to invoke fear or hope.

## 8 Data analysis and findings

No doubt, the spread of COVID-19 across the globe sends a chill down the spine of the entire humanity. This experience is the same in the Kingdom of Saudi Arabia. Consequently, there is fear representation in the reportage of the pandemic in order to make residents aware of the enormity of health threat posed by it. Three strategies are employed along this line: *authority, evidentiality and categorization*. Virtually all reports on the pandemic rely on statistics of new and cumulative infections as well as recoveries and deaths. The following are the instances of fear of ideological representations.

### 8.1 Ideological representation of fear

**Text 1 (Arab News, March 4, 2020)**

RIYADH: Saudi Arabia's health ministry announced on Wednesday a second case of coronavirus in the Kingdom.

The patient is a Saudi citizen who came from Iran via Bahrain and did not disclose that he had visited the country where more than 92 people have died according to official figures.

The citizen had accompanied the person who was reported on Monday as the first case of coronavirus in Saudi Arabia.

The ministry said the patient is in quarantine in hospital and that all those who have come into contact with him have been tested for coronavirus.

**Text 2 (Saudi Gazette, April 26, 2020)**

With *1,223 new coronavirus infections* recorded in Saudi Arabia during *the last 24 h*, the total number of cases now stand at *17,522,* the health ministry spokesman said on Sunday.

The ministry also recorded three new fatalities after one Saudi national and two non-Saudis died during the period, raising the death toll to *139*. The ages of the deceased range between 39 and 72 years.

An additional *142 people have recovered* within the past 24 h, raising *the total number of recoveries to 2,357*, Saudi Press Agency (SPA) quoted the ministry's spokesman Dr. Muhammad Al-Abdel Ali as saying.

**Text 3 (Saudi Gazette, April 29, 2020):**

Saudi Arabia recorded *1,325 new coronavirus cases* and *five new deaths* during the last 24 h, raising the total number of confirmed cases in the Kingdom to *21,402* and the death toll to *157*, the Ministry of Health announced on Wednesday.

Out of the newly reported cases, Saudi Arabian citizens make up only 15 percent. Meanwhile, 85 percent of the new cases were detected in non-Saudis, Saudi Press Agency (SPA) quoted the ministry spokesman Dr. Muhammad Al-Abdel Ali as saying.

Out of the *1,325 new cases* reported on Wednesday, *356 were confirmed in Makkah*, *225 in Madinah*, *224 in Jeddah*, and *203 in Riyadh*. There were *74 infections in Dammam, 42 in Hufof*, *40 in Jazan*, *37 in Buraidah*, *36 in Al-Khobar* and *23 in Jubail*.

Meanwhile, *2,953 people have recovered* from the coronavirus in Saudi Arabia. Authorities continue to urge people to stay at home unless necessary despite having relaxed some restrictions and curfews at the start of the holy month of Ramadan.

**Text 4 (Saudi Gazette, May 3, 2020):**

Saudi Arabia reported *1,552 new coronavirus cases* and *eight deaths*, bringing the total number of confirmed cases in the country to *27,011* and the virus-related death toll to 184, the Ministry of Health announced on Sunday.

Out of the newly detected cases, 19 percent are Saudi nationals and 81 percent are foreigners, Saudi Press Agency (SPA) quoted the ministry spokesman Dr. Muhammad Al-Abdel Ali as saying.

Sixteen 16 percent of the new cases are female and 84 percent are male, he added. The elderly make up only 2 percent of the new cases, children make up 5 percent, and adults make up 93 percent.

Eight people died in the last 24 h, including two Saudi citizens in Dammam and Jeddah, and six non-Saudis in Makkah, Riyadh, and Madina.

**Text 5 (Arab News, June 3, 2020)**

RIYADH: Saudi Arabia announced 30 more deaths from the novel coronavirus COVID-19 on Wednesday and 2,171 new cases of the disease.

Of the new cases, 683 were recorded in Riyadh, 418 in Jeddah, 279 in Makkah, 167 in Madinah and 133 in Dammam.

The health ministry also announced the recovery of 2,369 more patients, bringing the total number of recoveries in the Kingdom to 68,159.

A total of 579 people have died from the disease in the Kingdom so far.

**Text 6 (Arab News, June, 9, 2020, by Rawan Radwan)**

JEDDAH: More than 3,000 new cases of COVID-19 were confirmed in Saudi Arabia on Tuesday, and health authorities warned that the number is expected to rise in the coming days. The spike was blamed on failure to follow safety guidelines for public gatherings and social distancing rules.

Ministry of Health spokesman Dr. Mohammed Al-Abd Al-Aly said there had been ***a sharp rise in confirmed coronavirus cases in the past few days,*** as well as an increase in the number of patients admitted to critical care units. He added that 40 percent of the increase was the result of people in some parts of the country not taking the recommended precautions.

“Evaluation of the curfew will continue,” he said. “Some areas are adhering to precautionary measures and following the plans for the gradual return (to normality) — some are even advancing more than others.

While the death rate of 23 per million in Saudi Arabia remains significantly lower than the rates in many other countries, the Kingdom recorded its highest daily death count on Tuesday. There were 37 additional fatalities, raising the total to 783.

**Text 7 (Arab News, August 11, 2020):**

JEDDAH: ***Saudi Arabia reported 32 new coronavirus-related deaths on Monday, while the number of new cases in the Kingdom continues to fall.***

Cities across the country have recorded fewer than 100 cases per day in past weeks. The Kingdom recorded 1,257 new cases today, with Riyadh reporting 88, Dammam 65 and Hofuf 63.

Of the new cases, 83 percent are adults, with 5 percent over 65. In case numbers, 12 percent are children, while males make up 55 percent of today's confirmed cases.

***The total number of recorded cases in Saudi Arabia so far is 289,947. There are currently 33,270 active cases, 1,824 of which are receiving critical care***.

The above reports carve out fear by presenting statistics to provide evidence of COVID-19 threat, and the number of infection is often in thousands. Recording thousands of new infection daily calls for a sober reflection, as it may indicate possibility of many more undetected infections. It may also suggest rapid community transmission, indicating that residents are vulnerable to contract the virus if safety measures are not taken. These are the functions the statistics are ideologically meant to serve, and this constitutes the evidentiality strategy. Thus, in representing the ideology of fear, reports sufficiently rely on statistical evidence that are foregrounded and broken down to sub-categories.

i. With *1,223 new coronavirus infections* recorded in Saudi Arabia…, the total number of cases now stand at *17,522* …
ii. Saudi Arabia recorded *1,325 new coronavirus cases* and *five new deaths* …, raising the total number of confirmed cases in the Kingdom to *21,402* and the death toll to *157…*
iii. Saudi Arabia reported *1,552 new coronavirus cases* and *eight deaths*, bringing the total number of confirmed cases … to *27,011* and … death toll to 184…
iv. *The total number of recorded cases in Saudi Arabia so far is 289,947.* There are currently *33,270 active cases*, *1,824 … are receiving critical care.*

The above excerpts of reports are evidence providers to the danger posed by COVID-19. They serve as a red flag to the yet-to-be-infected residents of Saudi Arabia so that they can take extra caution in safeguarding themselves and their families from contracting the virus. Since numerous new cases are recorded within a space of 24 h, evidence of which is provided by statistics, it is a clear indication that the virus lurks around every nook and cranny of the country. Statistics do not only show the possibility of contraction alone, it also indicates daily increase in the number of victims that succumb to the virus. Even though there is no indication that the number of people who die daily of COVID-19 is above the number of those who die of other ailments such as tuberculosis, diabetes, hepatitis, or the number of those who die naturally without any ailment, presenting the statistics of daily deaths of coronavirus makes the virus appear as a more life-threatening infectious disease/virus that should be avoided by all costs.

Evidentiality strategy overlapses with categorization strategy to emphasize the severity of the situation by offering sub classifications of the cases which, in news reports, are ment to be informative of the state of cases, yet loaded informations and presence or absence of sub-classifications are functional. The following eight categories are found in the above reports: new infections, new recoveries, new deaths, cumulative (number of) infections, (cumulative number of) recoveries, total number of deaths, total number of active cases, and total number of critical cases. In addition to its statistics, each of these categories, with the exception of new recoveries and cumulative (number of) recoveries, presents some level of negativity of health, which the readers are expected to fear. For instance, while most of the categories appear to be in polarity (i.e. new infection/new recovery, total number of deaths/total number of active cases), one (total number of critical cases) does not have its corresponding opposite— total number of mild cases. It may be commonsensical to infer that deducting the number of critical cases from the total number of active cases should give us the total number of mild cases. But ideologically, the function is to foreground the category that emphasizes fear. This is so because the reports can also be presented in the other way by ignoring the categorization of critical cases and displaying the number of mild cases. But this would have the intended purpose of fear that the reports are meant to achieve.

In addition, the category of new recovery is regularly represented. For instance, it is represented in Text 2& 3 (i.e. An additional *142 people have recovered…*), but not in Texts 2,3 and 6—only the cumulative recovery cases are mentioned in Text 2 (i.e. Meanwhile, *2,953 people have recovered* from the coronavirus). In the same vein, the number of critical cases is mentioned in Text 7 (i.e. *1,824 … are receiving critical care.*), but nowhere is the number of mild cases mentioned in any of the other Texts. The question here is: why do we have certain categories regularly represented while others (their perceived existential opposite) are either occasionally represented or unrepresented at all? The response to this question is that the reports are interested in sustaining the fear already established around the pandemic within the country. That is why they are more interested in flagging the categories of people that have been negatively affected by the virus. This will serve to heighten the tension and fear in the minds of the Saudi Arabia residents.

Sometimes, emphasizing a threatening event in order to promote fear amongst residents is done through a strategic ordering of threat and hope events as in (Text 5 and 7). In Text 5 reconstructing the news report, at the distributional structural level with the outset of deaths through the use of "*30 more* deaths"and the ending sentence " a total of 579 have died…. *So far*)and at the lexical level through the use of "more deaths … so far " invoke feelings of frustration and panic at the outset and end of the report and disseminate like 'who is next' Quantification of recoveries is reduced to the middle of the text to direct intended fear communication. While in the report below, (Text 7), the ordering is confusing to the reader, as in below.

Saudi Arabia reported *32 new coronavirus-related deaths* on Monday, while the number of new infected cases in the Kingdom continues to fall.

What makes this report count as a depiction of fear ideology is the foregrounding of death cases even though infection cases “continues to fall" as fact in the present tense. It would have been expected that the order of the events would be infection first and death later since that is the natural occurrence of events— the people that died from the virus were first infected. The order that the report upholds, therefore, seeks to sustain the level of fear rather than early hope of success against the pandemic.

Apart from this, in employing the strategy of ***categorization***, an improper segregation into the philosophy of “*we*” and “they” can be discerned in Texts 2-3-4 as the following excerpts show:

**Text 2**

The ministry also recorded three new fatalities after *one Saudi national and two non-Saudis* died during the period, raising the death toll to *139*.

**Text 3**

Out of the newly reported cases, *Saudi Arabian citizens make up only 15 percent.* Meanwhile, *85 percent of the new cases were detected in non-Saudis,* Saudi Press Agency (SPA) quoted the ministry spokesman Dr. Muhammad Al-Abdel Ali as saying.

- Authorities continue to urge people to stay at home.

**Text 4**

Out of the newly detected cases, *19 percent are Saudi nationals* and *81 percent are foreigners*, Saudi Press Agency (SPA) quoted the ministry spokesman Dr. Muhammad Al-Abdel Ali as saying.

In a bid to establish fear through categorization, there has been over-categorization resulting in what may be termed ‘miscategorization’. For instance, infected persons are, amongst others, further sub-categorized as Saudis and non-Saudis—a needless categorization. This sub-categorization reveals the ideology of ‘we’ and ‘they’ already ingrained in Saudi life, and therefore reflects in early reports on COVID-19. There is no basis for this categorization as the virus does not segregate between nationalities in its attack. Sub-categorization into other units (young/adults/old; male/female) is important in order to instill fear in the categories of people that are susceptible to contracting the virus because of the nature of their lives. Sub-categorization into cases according to cities is also relevant because it helps to discourage residents from traveling to cities with serious infection despite the lifting of lockdown. Such sub-categorizations are useful in sustaining the ideology of fear upon which the reports are based. However, the same cannot be said of sub-categorization into Saudis and non-Saudis since this can hardly bring about a social distance between citizens and non-citizens. Social distancing in large group (regardless of nationalities) is generally recommended as a strategy of living within the pandemic. It is no wonder then that Saudi authorities were criticized by World Health Organization for sub-categorizing the infected people into citizen/non-citizen, and the criticism had imposed them to discard these categories in the reports that came later.

With reference to the socio-economic profile of the labor force in Saudi Arabia, categorizing Covid-19 victims into Saudi and Non- Saudi is due to many factors; some are genetic, and others are socio-economic. The Kingdom is a metropolitan area where people from different cultures and ethnic backgrounds live and interact together. Some genetic characteristics are associated with a particular race or ethnicities. These genetic factors may contribute to the increasing rate of deaths among people who belong to a particular race due to lack of immunity. There are some other factors related to the socio-economic background of the Non-Saudi residents. Many of these people have a limited income. Accordingly, they live together in large numbers in order to save money and support their families outside the Kingdom. Another important factor is related to the customs and traditions associated with specific nationalities; intimacy and rejection of social distancing. In all cases, the classification is not intended to discriminate against the Non- Saudi residents; on the contrary, it aims to avoid the spread of the disease among them, on the one hand, and to help enhance and promote medical and scientific research.

Much of the tension concerning the earlier categorization of non/Saudi is due to the global media representing individual cases of mistreatments without truth-seeking in connection to the socio-economic and historical Saudi Arabia working labor profile, disseminating information impartially and ethically. Of the total Saudi population of around 34,218,000, 10.43 million (76% of the country's workforce) working in the public labor market and private sector are mostly "unskilled foreign workers" who constitute 74%compared to 26%for the Saudi labor (General Authority for Statistics-Saudi Arabia, 2020, Quarter 1). Since the discovery of oil and the urgent need for the labour force, millions of migrants filled in manual and service jobs and the majority were unskillful. Over the years, social, ethical issues of corruption and in such critical times of Coronavirus increased as the majority disobey healthcare regulations The blame is not to put totally on the expats as the weak legislation and leniency are held responsible. Though Western media accuse Saudi Arabia of oppressive labor regimes, expats acknowledge Saudi Arabia to maintain their civil and human rights before and during the pandemic (Chaudhury, 2020). Mitigating between media attack during the pandemic, the Saudi government exempts residents from entry/exit visa fees for those whose Iqama has expired, supports the private work sectors, free medical treatment (Al-Amir, 2021), KPMG International Entities (Nomber 18, 2020).

With respect to the strategy of ***authority***, statistics would be inconvenient in achieving the above functions without supporting them with verifiable sources. It is against this background that proof of daily infection is backed by the topmost health authorities in the country as in all the 6 Texts representing fear (Text 1-2-4-5-7). Attributing some weight to reports as well as to convince the audience about the seriousness and contagiousness of the virus, news reports cite and quote authorities. Authorities whether institutional or personal roles differ in fear-triggering news reports as in the first texts they lend themselves to action "imposing further restrictions and negotiating curfew" which build up fear and negative cognitive experiences of the readers, while in hope-creating texts, authorities express admiration of "the coronavirus measures taken by the Kingdom", Text 11. Citing authorities are repeated at leadt twice in a news text and only once in Text 5. Text 1 quote two authorities outlets, in-text reference to authorities are underlined in the news texts above.

**Text 1**

… *the Ministry of Health announced on Wednesday*.

… *Saudis, Saudi Press Agency (SPA) quoted the ministry spokesman Dr. Muhammad Al-Abdel Ali as saying*.

Relying on institutional and senior individual authorities is meant to show that the statistics are the official information of the ministry of health and its representatives. But it also indicates the possibility of more existing infections, yet to be accounted for the authority. In any case, backing the claim of the statistical data up with an authority serves to convince the readers of the authenticity of the information. It is to make them take the underlining representation of fear with enough conviction.

### 8.2 Ideological representation of hope

The outset of a lot of Saudi news reports on COVID-19, from May till August 2020, and few instances when dealing with covid-19 related quarantine-leave offer hope for the inhabitants. Due to early intervention measures of closing borders and flights, mosques, schools and most significantly lockdown, infection rate is said to have dropped since the lifting of lockdown. The following are some instances of reports that have the ideology of hope represented:

**Text 8 (Saudi Gazette, May 6, 2020)**

Saudi Arabia's Health Minister Dr. Tawfiq Al-Rabiah said on Wednesday that *the global death rate from COVID-19 is ten times higher than that of Saudi Arabia.*

Text 8 quotes Saudi Arabia's Health Minister 's statement (in italics) meaning SA Covid-19 cases are low compared to the rest of the world". Such inverted comparison though highly reassuring and renders the utmost cognitive experience of hope in the readers, yet it is insensible comparing between nations countries in death incidents for many reasons, one of which is that it is misleading to compare mortality cases across countries of different population size and different containment strategies.

**Text 9 (Arab News, May 19, 2020)**

By: Deema Al-Khudair

**JEDDAH**: Saudi Arabia on Tuesday recorded a rise in the number of patients who have recovered from COVID-19 to 31,634, up from 28,748 recorded a day earlier.

Total recoveries have surpassed the number of active cases, which stands at 27,891. Of the total cases, 251 are in critical condition.

In the daily press briefing, Health Ministry spokesman Dr. Mohammed Al-Abd Al-Aly stressed the importance of relying on reliable sources of information about COVID-19.

The number of COVID-19 cases appears to be slowing down in Saudi Arabia. Highlighting the proactive measures taken by the Kingdom, Al-Aly said health authorities have conducted 618,084 screening tests.

**Text 10 (Saudi Gazette, July 23, 2020)**

Saudi Arabia's *single-day coronavirus recovery numbers have been rising* steadily with *new infections dropping despite* the lifting of lockdown measures.

The Kingdom on Thursday ***recorded 3,092 additional recoveries*** while it ***detected*** only ***2,238 new coronavirus cases*** over the past 24 h, according to the health ministry.

The other new infections were ***recorded*** in different cities and provinces across Saudi Arabia.

**Text 11 (Saudi Gazette, July 30, 2020)**

The Director-General of the World Health Organization (WHO), Dr. Tedros Adhanom Ghebreyesu, commended Saudi Arabia on the steps it has taken to ensure the health and safety of pilgrims during this year's Hajj.

He praised the coronavirus measures taken by the Kingdom, noting that it is a perfect example of what countries could do in order to adapt themselves to the new situation and coexist with the virus and protect the lives of people.

**Text 12 (Arab News, August 14, 2020)**

The Saudi Ministry of Health announced on Friday that the *total number of recoveries* from the novel coronavirus disease (COVID-19) pandemic has reached 262,959, *with 2,566 patients recovering in the last 24 hours…*

Texts 7 to 12 consist of different shades of hope for Saudi residents. These range from increase in recovery cases to decrease in death cases that are brought to the foreground deliberately. The first discourse unit in Text 10 (Saudi Arabia's ***single-day*** coronavirus ***recovery numbers have been rising steadily*** with new infections ***dropping*** despite the lifting of lockdown measures), for instance, consists of five items that are relevant to the representation of hope. These are: *single-day, recovery number, have been rising, steadily,* and *dropping*. To start with, the report foreground the issue of recovery (*recovery numbers*) while backgrounding the infection case (*new infections*). But beyond foregrounding *recovery*, there is an emphasis placed on *single-day* in order to suggest that the number is huge, at least in comparison with new cases of infection. The emphasis also extends to the verbal group, *have been rising*, in order to indicate gradual and sustained success against the pandemic. What completes the thread of emphasis is the adverbial, *steadily*, which serves to show that there is continuity in the victory. By way of denigrating the remnant of the virus in Saudi Arabia, a non-finite verb, *dropping,* is employed in describing new infections. The representation of success through emphasis and demotion of the virus through backgrounding and non-finite verb constitutes part of evidentiality strategy in offering hope to the residents of Saudi Arabia.

In the reproduction of Text 9, the news reporter Alkhudair designed the “recovery” structure to construct an optimistic perceived cognition. The discourse unit ***rise in number of patients who have recovered from Covid-19*** is foregrounded and also the second unit ***Total recoveries have surpassed the number of active cases***. Besides, discourse units that interrupts the positivity of evidentiality “***251 are in critical condition***” is immediately supported by an evaluative statement issued by the Ministry of Health spokesman declaring “***cases appears to be slowing down in Saudi***” and justify the state of the art due to ***proactive measures*** fostered by an institutional authority. In doing so, the news reporter direct the intended hope cognition of the public.

The reassurance/hope offers in (Text 10) hinges upon the increase in recovery rate and decrease in infection rate. While this does not really suggest the gradual end of the pandemic, it does offer light regarding it. That is, this gives the populace some confidence that Saudi authority has the capacity to rout the pandemic. The verb group, *have been rising*, used in reporting the single-day recovery number is worth noting. The aspectual marker of the verb group, *-en* and *–ing*, makes a combination perfect progression, an indication of continuity of this trend of recovery. Apart from this, the verb group is also of a finite type, which accords the value of importance to the entire clause where the verb group appears (*single-day coronavirus recovery numbers have been rising*), making it an alpha clause. On the contrary, the verb group, *dropping*, in the clause that reports the drop-in infection rate is of less importance when compared to *have been rising* since the former is an infinitive despite its marked progression. Thus, the entire clause where the infinitive verb appears (*new infections* ***dropping***) becomes a beta clause, and less in rank with the preceding clause. Thus, the ordering in this case foregrounds hopes contrary to what is found in the preceding section where the ideology of fear is foregrounded.

A similar situation is found in Text 12 except that the opening of the report does not even bother to accommodate the infection rate whatsoever: *the total number of recoveries from the novel coronavirus disease (COVID-19) pandemic has reached 262,959, with 2,566 patients recovering in the last 24 h.* Focusing on recovery incidents alone serves the purpose of giving hope of triumph against the virus to Saudi residents. The choice of the perfective aspectual verb group, ***has reached***, to report the cumulative recoveries has an indication of recognition of some level of success, and that gives the reason why the new recovery rate is less emphasized with the choice of infinitive progressive verb, *recovering*.

In terms of the discourse strategy employed in giving hope to the populace, evidentiality is often relied upon so that doubts are cleared off. Use of this strategy can be found in Texts 10 and 12. Text 9 provides statistical evidence to support the earlier claim of rising number of single-day recoveries (*recorded 3,092 additional recoveries*) and the dropping rate of new infections (*detected* only *2,238 new coronavirus cases*). The two sets of statistics presented above relate to recovery and infection rates respectively. While a single day recovery rate surpasses a single day infection rate by about a thousand, it is very important to pay attention to the reporting verbs of the two events. Whereas the event relating to recovery is reported with the verb, “recorded’, the one relating to infection is reported with “detected”. “Recorded” has an implication of victory, suggesting that the pandemic is an enemy to the kingdom. It is similar to reporting a football match score line in which a winning team could be said to record three goals against its opponent. Where “recorded” appears in the context of infection in the report, the verb is weakened through passivization as obvious in the following: *The other new infections were* ***recorded*** *in different cities and provinces across Saudi Arabia*. Apart from the fact that “recorded”, here, occurs in a passive clause, there is also the absence of evidentiality (of statistics) or of authority. This further weakens the force (of victory) inherent in the verb. On the other hand, “detected” has an implication of discovery, suggesting that the (pandemic) enemy is not visible, and that extra efforts are taken in unraveling its existence— another victory for the kingdom.

In addition to the evidentiality strategy (and the strategic verbs used), the report also provides a backing authority for its claim— *according to the health ministry*. This constitutes a supporting strategy of authority, which gives weight to the ideology of hope inherent in the report. This strategy is employed in several reports as in the underlined news texts in Text 8-11-12.

These underlined excerpts above do not only suggest hope of triumph over COVID-19, but they do this by providing strong authorities to represent this ideology. The reports either rely on institutional authority (such as *The Saudi Ministry of Health*) or both institutional and senior individual authorities (such as *Saudi Arabia's Health Minister Dr. Tawfiq Al-Rabiah; The Director-General of the World Health Organization (WHO), Dr. Tedros Adhanom Ghebreyesu;* and *Ministry of Health spokesman Dr. Mohammed Al-Abd Al-Aly*).

These institutional and senior figures authorities wield some kind of discourse power that gives their statements some weight or sanctity. Thus, their pronouncements are seen as almost infallible as they are expected to be a result of research. All the statements in the excerpts, therefore, point to the fact that Saudi government's response to the pandemic yields positive result. In Text 11, for instance, the praise showered on Saudi Arabia authority by the president of World Health Organization is an endorsement of the country's efforts against the virus, and therefore a line of hope for the residents. This suggests that the claims of gradual triumph by Saudi authority has been endorsed by a higher authority— a world authority on the issue of health endorsing a country authority. Relying on these authorities is a strategy by the news outlets to offer Saudi Arabia residents hope and tranquility of mind.

## 9 Conclusion

This study has examined the ideological representation of fear and hope in online newspaper reports on COVID-19 in Saudi Arabia. The findings show that three strategies are employed to represent the ideology of fear: authority, evidentiality and categorization. Most of the reports on COVID-19 heavily rely on statistics of new and cumulative infections as well as recoveries and deaths. The function of these statistics is ideologically employed to serve constitutes the evidentiality strategy. Evidentiality strategy often overlapses with categorization strategy as proof of the threat of the virus is broken down into units, each with its features and evidence. With respect to authority strategy, statistics would be inconvenient in achieving the above functions without supporting them with verifiable sources. This is why proof of daily infection is almost always backed up by the topmost health authority in the country.

Similarly, in giving hope to the populace, two strategies of evidentiality and authority are often used in Saudi online newspapers. Evidentiality strategy is employed upon so that doubts are cleared off through statistics of recovery cases particularly when a single day recovery rate surpasses a single day infection rate. Sometimes, choice of verbs in reporting recovery and infection cases plays a role in evidentiality strategy. Apart from this, there is also reliance on authority strategy in offering hope to the populace of the country in order to give some weight to the ideology inherent in the report.

In conclusion, ideologies of fear and hope on Covid-19 online news reports in Saudi Arabia are indirectly represented through the machinery of three strategies of evidentiality, categorization and authority. All the three strategies are complementary in the case of fear representation, but the strategy of categorization appears to be rarely used in the representation of hope. The study benefited from the ongoing contribution of Van Dijk in relation to the workings of ideology in discourse linguistic redesign in Covid-19 ideologies of fear and hope, yet macro and superstructure analysis would definitely shed light on the interplay between language, society and content writers. The present study is limited to twelve COVID-19 news reports investigated in terms of the strategies employed in the construction of ideologies; more extensive data may reveal different ideologies pertaining to coronavirus discourse communication cross-culturally on the expression of hope/fear in the Saudi context. Further studies in relation to the pragmatics of news reports macrostructures, the mircorules mediating between micro and macrostructures of covid-19 text interpretation, and metatextuality in news reports.

## Declarations

### Author contribution statement

Naimah Ahmad Al-Ghamdi: Contributed to the investigation, development and writing of this article.

### Funding statement

This research did not receive any specific grant from funding agencies in the public, commercial, or not-for-profit sectors.

### Data availability statement

Data included in article/supplementary material/referenced in article.

### Declaration of interests statement

The authors declare no conflict of interest.

### Additional information

No additional information is available for this paper.

## Figures and Tables

**Table 1:** The Data of the study.

“Saudi Gazette” 6 News Reports of Covid-29 Fear 3 Texts/Hope 3 Texts	“Arab News” 6 News Reports of Covid-29 Fear 4 Texts/Hope 2 Texts
∗ Saudi Gazette, May 3, 2020 https://saudigazette.com.sa/article/592734/SAUDI-ARABIA/Death-rate-from-coronavirus-in-Saudi-Arabia-very-low-says-Al-Rabiah ∗ Saudi Gazette, July 23, 2020 https://saudigazette.com.sa/article/595876/SAUDI-ARABIA/Recoveries-continue-to-surge-in-Saudi-Arabia-as-new-COVID-19-cases-drop ∗ Saudi Gazette, July 30, 2020 https://saudigazette.com.sa/article/596152/SAUDI-ARABIA/WHO-chief-praises-Saudi-Arabia-for-coronavirus-measures-during-Hajj ∗ Saudi Gazette, April 26, 2020 =https://saudigazette.com.sa/article/592360/?utm_redirect=301 ∗ Saudi Gazette, April 29, 2020 =https://saudigazette.com.sa/article/592457/?utm_redirect=301 ∗ Saudi Gazette, May 3, 2020 =https://saudigazette.com.sa/article/592639/?utm_redirect=301	∗ ArabNews, March 4, 2020 https://www.arabnews.com/node/1636926/saudi-arabia ∗ Arab News, May 19, 2020 https://www.arabnews.com/node/1676971/saudi-arabia ∗ Arab News, June 9, 2020 https://www.arabnews.com/node/1687141/saudi-arabia ∗ Arab News, June 3, 2020 https://www.arabnews.com/node/1684276/saudi-arabia ∗ Arab News, 11 August, 2020 https://www.arabnews.com/node/1717511/saudi-arabia ∗ Arab News, 14 August, 2020 https://www.arabnews.com/node/1719366/saudi-arabia
